# A central role of sulcal width in the associations of sleep duration and depression with cognition in mid to late life

**DOI:** 10.1093/sleepadvances/zpae058

**Published:** 2024-08-10

**Authors:** Caroline Faucher, Léonie Borne, Anna Behler, Bryan Paton, Joseph Giorgio, Jurgen Fripp, Renate Thienel, Michelle K Lupton, Michael Breakspear

**Affiliations:** School of Psychological Science, College of Science, Engineering and the Environment, University of Newcastle, Australia; Australian eHealth Research Centre, CSIRO, Brisbane, Australia; School of Psychological Science, College of Science, Engineering and the Environment, University of Newcastle, Australia; School of Psychological Science, College of Science, Engineering and the Environment, University of Newcastle, Australia; School of Psychological Science, College of Science, Engineering and the Environment, University of Newcastle, Australia; School of Psychological Science, College of Science, Engineering and the Environment, University of Newcastle, Australia; Helen Wills Neuroscience Institute, University of California, Berkeley, USA; Australian eHealth Research Centre, CSIRO, Brisbane, Australia; School of Public Health and Medicine, College of Health Medicine and Wellbeing, University of Newcastle, Australia; Genetic Epidemiology, QIMR Berghofer Medical Research Institute, Queensland, Australia; School of Psychological Science, College of Science, Engineering and the Environment, University of Newcastle, Australia; School of Public Health and Medicine, College of Health Medicine and Wellbeing, University of Newcastle, Australia

**Keywords:** sleep duration, depression, aging, cognition, brain health, sulci width

## Abstract

**Study Objectives:**

Evidence suggests that poor sleep impacts cognition, brain health, and dementia risk but the nature of the association is poorly understood. This study examined how self-reported sleep duration, napping, and subjective depression symptoms are associated with the brain-cognition relationship in older adults, using sulcal width as a measure of relative brain health.

**Methods:**

A canonical partial least squares analysis was used to obtain two composite variables that relate cognition and sulcal width in a cross-sectional study of 137 adults aged 46–72. We used a combination of ANCOVA and path analyses to test the associations of self-reported sleep duration, napping, and subjective depression symptoms with the brain-cognition relationship.

**Results:**

We observed a significant main effect of sleep duration on sulcal width, with participants reporting 7 hours showing narrower sulci than other durations. This effect remained significant after including subjective depression as a covariate, which also had a significant main effect on sulcal width in the model. There was no significant effect of napping on sulcal width. In path analyses where the effects of age, self-reported sleep duration and depression symptoms were investigated together, sulcal width mediated the relationship between age and cognition. We also observed a significant indirect effect of sulci width in the subjective depression-cognition relationship.

**Conclusions:**

Findings suggest that self-reported sleep duration and subjective depression may each be independently associated with brain morphology, which is related to cognitive functions. Results could help inform clinical trials and related intervention studies that aim at delaying cognitive decline in adults at risk of developing dementia.

## Graphical Abstract

**Graphical Abstract:**
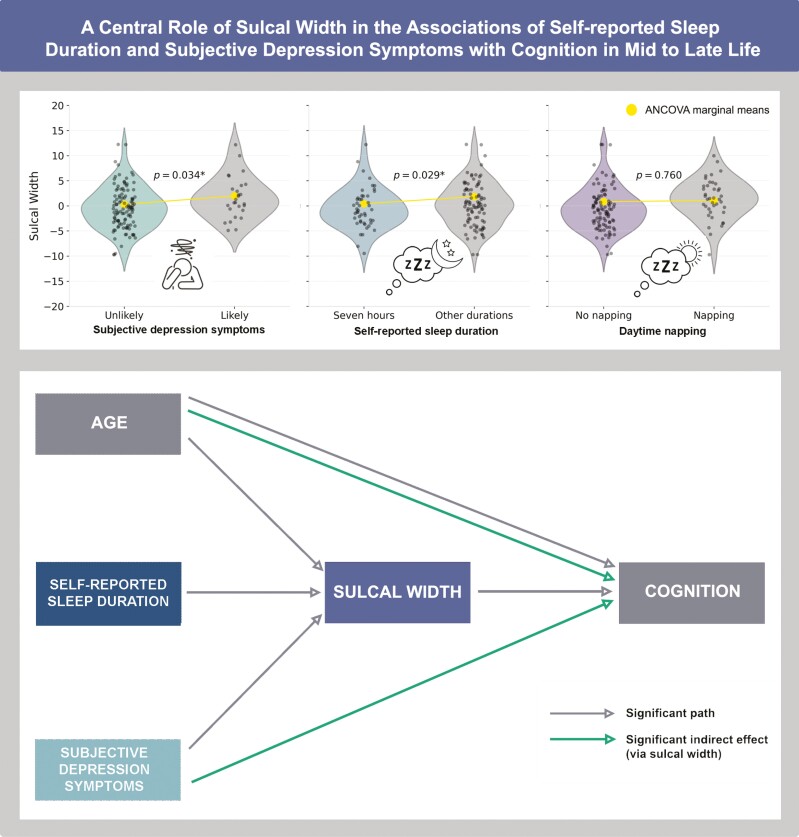

**Statement of Significance**

Sleep and depression have been identified as potentially modifiable factors for dementia risk. Yet, their interrelationships with brain health and cognition are still poorly understood. Our study contributes to bridging this gap by highlighting potential independent associations of self-reported sleep duration and subjective depression with sulcal width, a sensitive marker of brain morphology, which is linked to cognitive function in older adults. This could help inform clinical trials and related intervention studies that aim at delaying cognitive decline in adults at risk of developing dementia.

The aging population highlights the need to understand and promote quality of life and wellbeing in old age. Substantial evidence exists for several protective lifestyle factors for cognition in aging [1]. While sleep is not included in Livingston’s life-course model of modifiable risk factors for dementia, mounting evidence points to the role of adequate sleep in maintaining cognitive abilities in later life [2, 3]. For example, people with better sleep attributes tend to have superior cognitive performance and a reduced risk of developing dementia in later life [4–6].

Among the various sleep attributes, overnight sleep duration has been extensively studied. It is well established that total sleep time declines from childhood to old age [7], with adults older than 60 sleeping for approximately eight hours of sleep per night, down from 10 and half hours in early adulthood [8]. Previous research focused on the extremes of sleep duration has found that both short and long sleep durations are associated with poorer cognitive function [9, 10]. Recently, a non-linear relationship between cognition and sleep duration has been suggested. A large study using UK Biobank data found quadratic associations between self-reported sleep duration and various cognitive abilities, including processing speed, visual attention, memory, and problem-solving skills [11]. Approximately 7 hours of overnight sleep was found to be the optimal duration. Ma et al. [12] also observed an inverted U-shaped association between self-reported sleep duration and global cognitive decline. In the longitudinal analysis, the cognitive scores of individuals with ≤4 hours or ≥10 hours of self-reported overnight sleep declined faster than in the reference group who reported 7 hours of sleep. These findings align with an earlier meta-analysis that examined U-shaped associations between sleep duration and the risks of cognitive decline, mild cognitive impairment, and dementia, which also indicated that an average sleep duration of 7 hours provided the greatest cognitive benefits [13].

In addition to its association with cognitive functions, overnight sleep has also been linked to brain health using structural magnetic resonance (MRI) imaging. Older adults who report inadequate sleep tend to have smaller cortical volume [14]. Other findings specific to sleep duration suggest that the optimal sleep duration for maintaining brain health in older age ranges from six to eight hours of sleep [15]. In turn, it is well accepted that structural changes in the brain correlate with cognitive abilities [16] with cortical atrophy associated with poorer performance. However, research into the relationships between sleep, brain health and cognition has tended to focus on specific cognitive functions or brain measures, without considering their interrelationships. Additionally, few studies have accounted for other risk factors that may co-exist with sleep disturbances, and may also contribute to atrophy or poor cognition, such as daytime napping and low mood.

Daytime napping habits are linked to negative nocturnal sleep outcomes in older adults [17] and were identified as a bidirectional factor for dementia [18]. Like sleep duration, napping is shaped by biological and environmental factors. In countries such as the United States, where daytime napping is not traditionally practiced, 75 to 84-year-old adults are more than twice as likely to take regular naps compared to those aged between 55 and 64 years old [19]. The exact role of napping in late life is yet to be fully understood and evidence on how it may influence cognitive abilities is mixed. Some studies report benefits on cognitive performance [20], while others suggest that extended napping duration is linked to poor performance [21].

Late-life depression has been identified as a key risk factor for dementia, with adults over the age of 65 with depression being almost twice as likely to develop the disease [1]. This form of depression is often considered as a distinct subtype, with unique underlying etiologies, resulting in difference in symptomology [22]. Consequently, many studies investigating late-life depression in the context of cognitive decline utilize self-reported measures specifically designed for the geriatric population. The Geriatric Depression Scale 15, for instance, has been described as an effective tool in differentiating between minor and major depressive disorder in the older adults specifically [23].

In this demographic, depressive symptoms are also related to sleep disturbances [24]. Few studies have specifically investigated sleep duration as a particular parameter in this relationship with depression. However, a recent meta-analysis of middle-to-older-aged adults suggests that both short and long sleep durations are associated with an increased risk of depression compared to an average of 7 hours of sleep [25].

Evidence suggests a complex interplay among sleep, depression, and cognition in older adults. Both sleep disturbances and depression have been found to mediate the relationship between age and mild cognitive impairment [26]. In a study looking specifically at sleep duration in a sample aged 45 and over, depressive symptoms mediated the relationship between self-reported sleep duration and cognitive function. Still, the mechanisms underlying the intricate connections among sleep, depression, and cognitive function in older adults remain partially understood. However, this underscores the necessity of considering depressive symptoms when investigating the sleep-cognition relationship.

The aim of the present study is to disambiguate the associations of self-reported sleep duration with the brain-cognition relationship in older adults using sulcal width as a measure of cortical anatomy. Sulci width, derived from MRI, has been shown to be a sensitive measure of cortical changes in the older population [27]. In contrast to other MRI techniques such as cortical thickness, the extraction of sulcal morphology is unique in that it does not rely on the contrast between white and grey matter, which typically diminishes with age [27]. We hypothesize that the brain-cognition relationship is associated with self-reported sleep duration, both overnight and daytime. Given the evidence of an interplay among sleep duration, depression, and cognition in older adults, we also hypothesize that the brain–cognition relationship is related to subjective depression symptoms. We quantify the brain–cognition association through the use of a multivariate brain-behavior technique called partial least squares (PLS). We use a combination of analyses of covariance and mediation analyses with a path analysis to test the associations of self-reported sleep duration, daytime napping, and subjective depression symptoms with the brain-cognition relationship.

## Methods

Ethics approval was obtained from the Human Research Ethics committees of QIMR Berghofer (P2193 and P2210), the University of Queensland (2016/HE001261), The University of Newcastle (H-2020-0439) in accordance with the National Statement on Ethical Conduct in Human Research.

### Participants

Participants were drawn from the Prospective Imaging Study of Ageing: Genes, Brain and Behaviour (PISA [28];). PISA is a large prospective cohort study of midlife and older Australians with low and high genetic risk of developing dementia. Participants were previously recruited from the general population as part of extensive in-house cohorts from GWAS studies and studies of twins and their families. Participants were not excluded based on any health conditions, including mental health disorders such as major depressive disorder or sleep disorders. The subsample for this study included participants aged 46–72 years who completed online and in-person cognitive assessments, questionnaires, and imaging. To be included in the present study, participants required cognitive, sleep, mood, and MRI data.

### Subjective depression symptoms

The Geriatric Depression Scale 15 (GDS-15 [29];), a self-reported questionnaire, was used to assess depression symptoms. The GDS-15 is a short version of the original scale developed as a screening instrument for older adults specifically [30]. We used a cutoff of ≥5 for our categories (i.e. normal/subjective depression) based on the following scoring recommendation [31]: 0–4 (normal), 5–9 (mild), and 10-15 (moderate to severe).

### Self-reported sleep assessment

Sleep parameters were assessed using self-reported online questionnaires. Sleep quality was calculated from the Pittsburgh Sleep Quality Index (PSQI [32];). The PSQI assesses 7 sleep domains: sleep quality, latency, duration, efficiency, problems, medication, and daytime tiredness. The global score ranges from 0 to 21, with the higher number indicating poorer sleep quality. Participants with a PSQI scores of ≤5 were coded as having good sleep quality, while those with >5 were coded as poor sleep quality (see Table 1). Sleep duration was derived from question 4, “During the past month, how many hours of actual sleep did you get at night?.” As a constraint of the PISA multi-choices questionnaire, participants had to round their duration to the nearest 30 minutes, which ranged from <4 hours to >12 hours. Sleep duration was further segmented into two categories: seven (including 7–8 hours) versus other (<7 or ≥8 hours). This aligns with recent research classification suggesting a U-shaped association of sleep duration with brain health and cognition [11–13, 15], which are our outcomes of interest.

**Table 1.** Participant demographics, depression, and sleep characteristics

Demographics (*n = *137)	
Age	
Range	[46–72]
Mean	60.4 ±* *5.96
Female, *n* (%)	104 (76%)
*APOE* allele combinations, *n* (%)	
*ε*2/*ε*3	8 (5.8%)
*ε*2/*ε*4	6 (4.4%)
*ε*3/*ε*3	54 (39.4%)
*ε*3/*ε*4	61 (44.5%)
*ε*4/*ε*4	8 (5.8%)
Education, year	14.37 ± 2.45
Geriatric depression scale (GDS)	
Range	[0-13]
Mean	2.93 ± 2.97
Total likely depressed, *n*	20
Self-reported sleep	
PSQI global score (*n = 135)*	
Range	[0,14]
Mean	5.23 ± 3.05
Good/poor sleep quality (*n*)	83/52
Sleep duration, hour	6.97 ± 1.11
< 7 hours, *n*	57
7 hours, *n*	47
≥8 hours, *n*	33
Napping (no/yes), *n*	97/40

Data are mean ± SD. Total *n *= 137 except when specified. GDS scores 5 and above indicate participants are likely depressed. PSQI scores 5 and below are associated with good sleep quality and over 5 are associated with poor sleep quality.

Another questionnaire assessed daytime napping habits using the multiple-choice question “How many hours do you spend napping in a typical day?.” Answers ranged from none to >6 hours, with choices rounded to the hour in between. We further categorized napping into no napping versus napping.

### Cognitive assessment

Cognition was assessed using the self-administered online battery Creyos (formerly Cambridge Brain Sciences). The Creyos battery consists of 12 neurocognitive tasks assessing multiple cognitive domains including memory, reasoning, verbal ability, and concentration (see Supplementary Appendix A for a full description of each test. More details can be found at Creyos.com). The online assessment took approximately 30 minutes to complete. Cognitive scores were recoded so that a higher positive value indicated better performance, while a lower value indicated worse performance (e.g. error rate, reaction time). All 12 tests were included in subsequent statistical analysis to obtain a composite variable of cognition (i.e. partial least squares analysis described later).

## APOE

*The* *apoliproprotein E (APOE)* genotype (*ε*4 allele carriers vs non-carriers) was determined from blood-extracted DNA using TaqMan SNP genotyping assays on an ABI Prism 7900HT and analyzed using SDS software (Applied Biosystems). Participants who were carriers of at least one *APOE ε4* gene were categorized as *APOE ε4* positive.

### MRI imaging data

Structural imaging data was acquired using a T1-weighted 3D MPRAGE sequence (TE/TR = 2.26 ms/2.3 s, TI = 0.9 s, FA = 8˚, 1 mm isotropic resolution, matrix 256 × 240 ××192, BW = 200 Hz/Px, 2× GRAPPA acceleration) on a 3T Siemens BioGraph mMR. Other MRI modalities including functional, diffusion, and spectroscopy sequences were acquired but are not analyzed in the present study [28].

### Data processing and modeling

Sulcal width was extracted using the Morphologist pipeline of the BrainVISA toolbox, which identifies 127 cortical sulci [33, 34]. Left and right hemisphere measurements were averaged when the same sulci were present in each hemisphere, resulting in 64 unique measurements (see Supplementary Figure S2 for full labels). Sulci missing in more than 50% of participants were not used in subsequent analyses.

### Statistical analyses

#### Partial least squares

**Figure 1.**
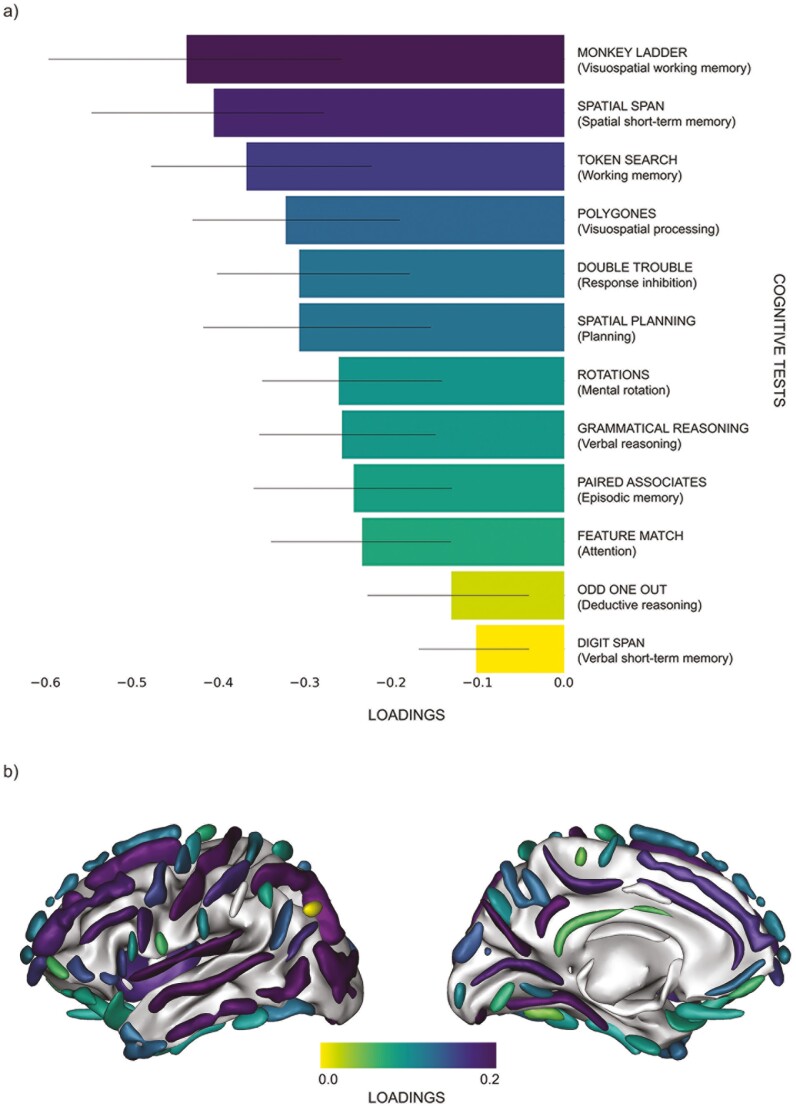
Representations of loadings from partial least squares analysis. Loadings of cognitive tests (a) and cortical sulci (b) contributing to the principal brain-behavior mode. A high loading (close to 1 or −1) suggests that the original observed variable contributed considerably to the composite variables, in this case either to the cognition or sulci width variables. In contrast, a loading close to 0 indicates a negligible contribution to the composite variables. Black bars represent confidence intervals. (c) Visual representation of sulci based on loadings illustrated in figure (b) where a darker shade indicates a stronger contribution of that sulcus to the sulci width composite variable. Sulci width atlas with full anatomical names of acronyms and their location can be viewed in Supplementary Figure S2.

We performed a Canonical PLS analysis in Python as a data reduction method to extract one composite variable per dataset; one of cognition and one of sulci width. All 12 cognitive subtest variables and all sulci width variables were entered into this analysis (excluding sulci variables missing values for >50% of the participants). The PLS returned one composite variable of sulci anatomy (i.e. sulcal width) and one composite variable of cognition, while maximizing the covariance between the two datasets. This multivariate regression method produces several modes of brain-behavior co-variation from the observed variables, with each mode comprising a weighted composite of the individual scores. Permutation tests, repeated 1000 times, assessed the robustness of each mode. The contribution of each individual sulcus and cognitive test to the shared variance is reflected as loadings contributing to a mode (see example in Figure 1). In the context of PLS analysis, each mode corresponds to a pair of composite variables, one from each set of observed variables. A high loading (close to 1 or −1) suggests that the original observed variable contributed considerably to the composite variable. In contrast, a loading close to 0 indicates a negligible contribution. The composite variables from the selected mode, in this case one for sulci width and one for cognition, were used in subsequent analyses, including in the analyses of covariance (ANCOVA) and path analyses described below.

#### ANCOVA

First, we investigated the association between self-reported sleep duration and cognition, as well as the association between self-reported sleep duration and sulcal width, with sex, and age as covariates. We then added subjective depression as a covariate of interest in the two ANCOVA above. The relationships between daytime napping and each composite variable (cognition and sulcal width) were also measured by ANCOVA with sex and age as covariates. We ran an additional ANCOVA (see Supplementary Table S1) where short and long sleep durations were further split into two categories, resulting in a total of three categories: <7 hours, 7 hours, ≥8 hours. We also ran an ANCOVA between our dependent variables and *APOE* ε4 status (positive or negative), with sex and age as covariates. An alpha level of 0.05 was used for the null hypothesis significance testing.

#### Chi-square test of independence

A chi-square test of independence was employed to investigate if there was a relationship between sleep duration and depression likelihood.

### Model analyses

We employed mediation and path analyses, using JASP, to further understand the relationships from the ANCOVAs results. We first executed a simple mediation model in which an indirect relationship can be established between a predictor and outcome [35]. Age was added as the independent variable in the model with cognition as the dependent variable and brain health (i.e. sulcal width) as the mediator. For the second model, sleep duration and depression likelihood were added to the simple mediation model as predictors, along with age, to further understand their role in the relationship between sulci widening and age-related cognitive decline.

## Results

The original subsample included 163 PISA participants. The dataset was inspected to ensure only one person per pair of twins was included in analyses. After removal of participants with more than 50% data missing in sulci width measurements and of two outliers, the final sample consisted of 137 participants (see **Table 1**). These participants were included in further statistical analyses.

Age, sex, years of education, and subjective depression symptoms were not significantly different between those who slept 7 hours and those reporting other durations.

### Composite variables of cognition and sulcal width

Three brain features with more than 50% of values missing across all participants were dropped from the PLS analysis: the anterior sub-central ramus of the lateral fissure, the sulcus of the supra-marginal gyrus, and the intracingular sulcus. No cognitive tests were excluded.

In our study, the PLS returned one robust mode of covariation between sulcal width and cognition (1st mode, *p* < .001, cov = 3.07, *z*-cov = 5.47, *r*^2^ = 0.17; 2nd mode, *p* = 1.00). Working memory and short-term memory contributed strongest weights to the cognitive loadings (Figure 1a). The posterior inferior temporal sulcus, the occipital lobe, and the intraparietal sulcus contributed the strongest weights to the cortical loadings (Figure 1b). Composite variables used in the study were derived from this 1st mode of covariation.

### Effects of sleep measures and depression likelihood on composite variables

**Figure 2.**
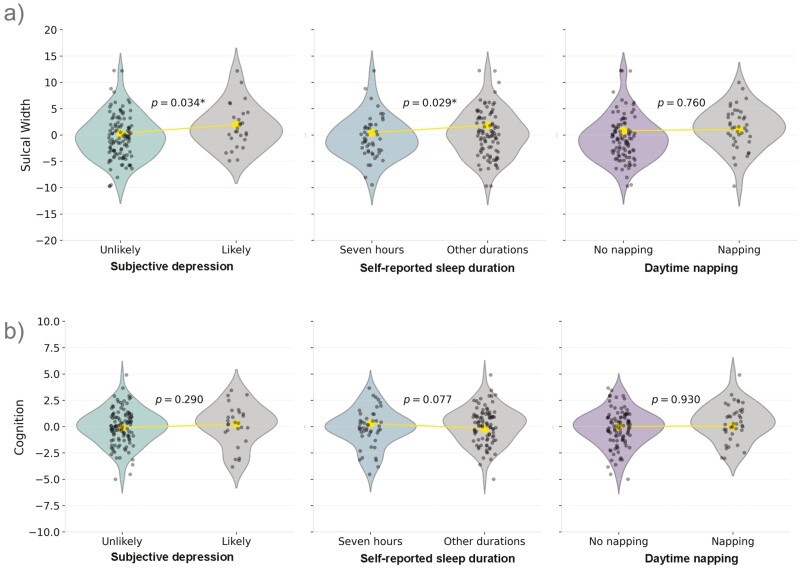
Main effects of sleep, and depression on sulci width and cognition composite variables. Difference in sulcal width (a) and cognition (b) by group; depression likelihood, sleep duration and daytime napping. Violin plots show distribution. The large dot in each the violin plot indicates the marginal mean. *p ≤ .05.

ANCOVA analyses showed differences in sulcal width between self-reported sleep duration and subjective depression groups, with small to moderate effect sizes (Figure 2; Table 2). We observed a significant main effect of sleep duration on the sulcal width composite variable (*F*(1, 133) = 4.17, *p* = .043, partial *η*² = 0.03), with age and sex as covariates. This effect remained significant (*F*(1,132) = 4.89, *p* = .029, partial *η*² = 0.04) after including depression likelihood as a covariate, which was also significant in the model (*F*(1,132) = 4.62, *p* = .034, partial *η*² = 0.03). Participants who reported sleeping less than 7 or 8 or more hours possessed wider sulcal width (marginal mean = 1.84, SE = 0.47) than those who slept 7 hours (marginal mean= 0.42, SE = 0.57). Participants with subjective depression had wider sulci (marginal mean = 1.97, SE = 0.69) than those without (marginal mean = 0.30, SE = 0.42). The effect of napping (*F*(1,133) = 0.09, *p* = .760, partial *η*² = 7.045 × 10^−4^) on sulcal width was not statistically significant. Contrary to our hypothesis, the chi-square test indicated that the association between subjective depression and self-reported sleep duration was not significant (*χ*^*2*^ = 0.31, *p* = .58), suggesting that these two factors may be independently related to sulcal width.

**Table 2.** Results from ANCOVA aanalyses

	Dependent variable	Source	*F*	*P*	Partial *η*²
1.	Cognition (Covariates: age and sex)	Overnight sleep duration	2.93	.089	0.02
2.	Cognition (Covariates: age, sex, and depression symptoms)	Overnight sleep duration	3.17	.077	0.02
		Depression symptoms	1.13	.291	0.01
3.	Cognition (Covariates: age and sex)	Napping	0.01	.925	6.614 × 10^-5^
4.	Sulcal width (Covariates: age and sex)	Overnight sleep duration	4.17	.043*	0.03
5.	Sulcal width (Covariates: age, sex, and depression symptoms)	Overnight sleep duration	4.89	.029*	0.04
		Depression symptoms	4.61	.034*	0.03
6.	Sulcal width (Covariates: age and sex)	Napping	0.09	.760	7.045 × 10^-4^

*p ≤ .05. A total of six ANCOVA analyses were computed. Overnight sleep duration (7 hours/<7 or ≥8 hours), napping (yes/no), and depressive symptoms (normal/elevated) were assessed via self-reported questionnaires. Categories are further described in Method section.

In contrast to the effects on sulcal width, there was no significant main effect of any of the variables of interest on the cognition variable.

Additional ANCOVA showed no significant main effects of subjective sleep duration on the dependent variables when sleep durations were segmented into three categories (<7 hours, 7 hours, ≥8 hours; Supplementary Tables S1 and S2). The lack of a significant result for the sulcal width composite variable may be due to reduced statistical power. Also, there was no significant main effect of *APOE* ε4 status (positive or negative) on our dependent variables (Supplementary Table S3).

### Mediation analysis

**Figure 3.**
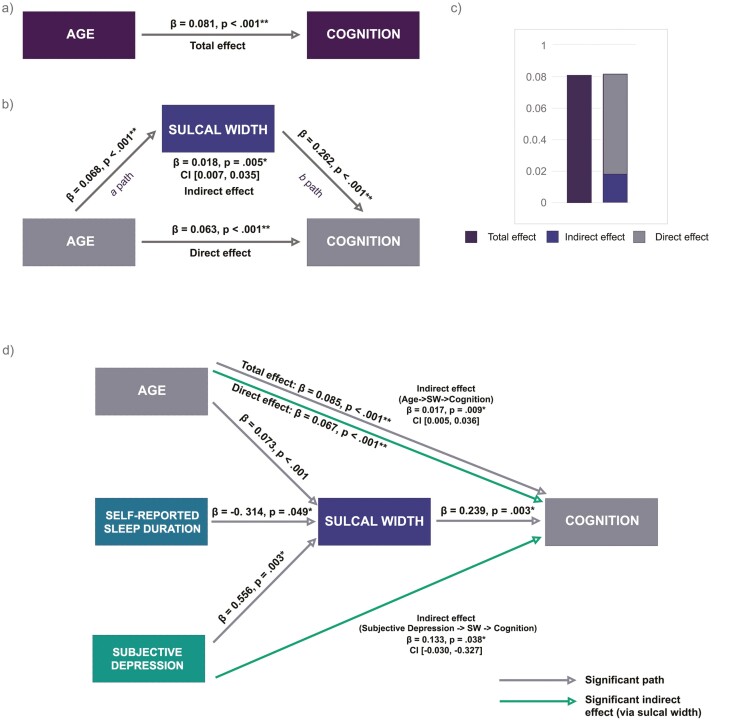
Mediation models with sulcal width as mediator. Model 1: (a) Total effect of the relationship between age and cognition, without mediator included in the model. (b) Mediation model representing direct effect of age on cognition, and indirect effect of sulcal width as a mediator. (c) Comparison of effects as a result of mediation analysis. Model 2: (d) Mediation model with age, sleep duration and depression as predictors. *p < .05, **p < .001. Mediation analysis, 5000 bootstrap samples and 95% confidence intervals (CI). *Β* indicates standardized values. CI from unstandardized beta coefficients. Continuous lines indicate significant associations.

Results of the base mediation model in this cross-sectional sample (Model 1 illustrated in Figure 3) showed a significant partial mediating effect of sulci width in the relationship between age as the predictor and cognition as the outcome (*β* = 0.018, *p* = .005, CI [0.007, 0.035]). All other paths in the model, including direct and total effects, were significant (Table 3). As expected, older age was associated with wider sulcal width and poorer cognition, reflected in positive beta coefficient values due to loadings from composite variable. Greater sulcal width was also associated with poorer cognitive performance.

**Table 3.** Mediation analyses with sulcal width as mediator and cognition as outcome

Model (Outcome)	Total effect	Direct effect	*a* path	*b* path	Indirect effect
Model 1					
A (age -> sulcal width -> cognition)	*β* = **0.081** *p* < .001**	***β* = 0.063** *p* < .001**	***β* = 0.068** *p* < .001**	***β* = 0.262** *p* < .001**	***β* = 0.018** *p* = .005*, CI[0.007, 0.035}
Model 2					
B (age -> sulcal width -> cognition	*β* = **0.085** *p* < .001**	*β* = **0.067** *p* < .001**	*β* = **0.073** *p* < .001**	*β* = **0.239** *p* = .003	***β* = 0.017** *p* = .009*, CI[0.005, 0.036}
C (sleep duration -> sulcal width -> cognition)	*β* = -0.283 *p* = .071	*β* = -0.208 *p* = .178	***β* = -0.314** *p* = 0.049	*β* = **0.239** *p* = .003	*β* = -0.075 *p* = .103 CI[−0.200, −0.008}
D (depression -> sulcal width -> cognition	*β* = 0.218 *p* = .241	*β* = 0.085 *p* = .647	***β* = 0.556** *p* = .003	*β* = **0.239** *p* = .003	***β* = 0.133** *p* = .038 CI[−0.030, −0.327}

*p ≤ .05, **p ≤ .001, 5000 bootstrap samples and 95% confidence intervals (CI). *Β* indicates standardized values. Bold beta values are significant. CI from unstandardized beta coefficients.

Adding sleep duration and depression likelihood to the model (Model 2; Figure 3) revealed a significant indirect effect via sulci width in the relationship between depression likelihood and cognition (*β* = 0.133, *p* = .038, CI [0.030, 0.327]). Neither the total nor direct effect of depression on cognition were significant. Furthermore, there was no significant mediating effect of sulcal width in the model with sleep duration as a predictor. However, 7 hours of overnight sleep was significantly associated with narrower sulcal width (*β* = −0.314, *p* = .049), which in turn was associated with better cognition (*β* = 0.239, *p* = .003).

## Discussion

We studied the complex associations of sleep and depression symptomology on the brain-cognition relationship in mid to late life. To the best of our knowledge this is the first study investigating the interplay of these factors together using sulcal width. Overall, our findings suggest that two potentially modifiable factors, subjective depression symptoms and self-reported sleep duration, may each be independently related to brain health in mid to late life, with small to moderate effect sizes observed. In our path analysis, we found a partial mediating effect of sulci width in the age-cognition association. We also found a significant indirect effect via sulci width in the relationship between subjective depression and cognition. Furthermore, 7 hours of overnight sleep was associated with narrower sulci width than other durations, which in turn was associated with better cognition.

These findings converge with a growing body of evidence that suggests a non-linear effect of sleep duration on brain structure. In the present study, sulci width was used as a brain morphology measure sensitive to age-related changes. This method is also suggested to be less susceptible to age-related contrast issues encountered with more volumetric MRI methods [27, 36]. We found that 7 hours of overnight sleep was associated with narrower sulci than other durations. This amount of sleep is within the range reported by other studies suggesting 6–8 hours to be the optimal overnight sleep duration for brain health [11, 15, 37]. The U-shaped was also qualitatively shown in (Supplementary Figure S1, where short and long durations were further differentiated.

While the mechanisms are not fully understood, one prominent view is that adequate sleep/wake cycles play a key role in the balanced production and clearance of amyloid (Aβ) and Tau proteins, and neurotoxic waste products. Therefore, inadequate sleep in older adults may disturb this balance, resulting in accumulation of Aβ and Tau in the brain [38]. Supporting this hypothesis, several studies have reported a robust association between poor sleep and a larger burden of Tau and Aβ [39, 40]. This may particularly be of interest in our sample where 55% of participants have at least an *APOE ε*4 allele, thus more at risk of developing dementia. Interestingly, Aβ accumulation may occur several years before any cognitive symptoms, which could explain why sleep duration was not significantly associated with cognitive performance in our sample. Alternatively, given that the composite variable loaded strongly on working and short-term memory, it may be that sleep duration is more strongly associated with other domains. For instance [11], found a non-linear association between sleep duration and visual attention using a large sample of the UK Biobank.

Our study suggests a potential role of brain structure in mediating the association between depression symptoms and cognition in mid to late life. This aligns with recent literature that suggests that the influence of depression on the brain may be mediated via changes in neurochemistry or brain plasticity, then leading to alterations in cognitive abilities (see [41]). Notably, high cortisol levels from a hyperactive hypothalamic–pituitary–adrenal axis are associated with major depressive disorder [42]. In turn, prolonged elevated cortisol levels are linked to atrophy to hippocampus, an area essential for learning and memory [43]. Alternatively, it has been proposed that social isolation, as a consequence of depression, could lead to loss of overall brain volume, and grey matter in the hippocampus [44].

A prior meta-analysis has reported an association of depression severity with cognitive function [45]. In the present study, we did not find a direct association between depressive symptoms and cognition. Again, this could be due to the cognition composite variable loading mostly on short-term and working memory. Our sample also reported somewhat low severity of symptoms, which may not have been high enough to affect cognition directly and could have contributed to the lack of significant direct association. We did, however, observe a significant association of depression likelihood and sulci width as well as a significant indirect association with cognition via sulci width. This may suggest a more subtle, or complex mechanism in our mid to late life cohort. Replicating the study in a cohort enriched for major depression disorder could provide additional insights into the impact of depression on brain morphology and cognition.

This study is not without limitations. Notably, sleep was assessed using subjective measures. As such, the results should be interpreted with caution. Self-reported sleep can give insights into a person’s perception of sleep. However, people generally overestimate sleep time when self-reporting sleep duration compared to objective sleep parameters measured by polysomnography [46, 47]. Future studies could use objective measure of sleep, in turn allowing for additional micro and macro architecture components to be investigated.

Although the sample is representative of the lifetime prevalence of depression in the general population [48], it is worth noting the majority of participants had low depressive symptoms. In addition, as there was no significant effect of *APOE* ε4 status (positive or negative) on our dependent variables, we did not use this genetic risk factor in further models. Future studies with larger numbers could explore this relationship specifically. It is also worthy to note that the study adopted a cross-sectional design. Therefore, results should be interpreted with caution. A longitudinal design would allow to test our hypothesis more robustly, providing clearer insights into the causal relationships involved. It would allow quantifying individual trajectories, and identification of more nuanced effects, such as resilience to cortical atrophy.

Beyond the statistical significance, which is supported by effect sizes varying from small to moderate, our findings hold potential translational significance. We have found that self-reported sleep duration and subjective depression may each be independently associated with brain morphology, which is related to cognitive functions. This could help inform the design of clinical trials and intervention studies aimed at delaying cognitive decline among adults at risk of developing dementia.

## Supplementary Material

zpae058_suppl_Supplementary_Tables_S1-S3_Figures_S1-S2

## Data Availability

Following completion of each wave (baseline, follow-up) and appropriate quality control, de-identified PISA data will be made available to other research groups upon request. Due to privacy, confidentiality and constraints imposed by the local Human Research Ethics Committee, a “Data Sharing Agreement” will be required before data will be released. Due to ethics constraints, data will be shared on a project-specific basis. Depending on the nature of the data requested, evidence of local ethics approval may be required.

## References

[1] Livingston G , Huntley J, Sommerlad A, et al. Dementia prevention, intervention, and care: 2020 report of the Lancet Commission. Lancet (London, England). 2020;396(10248):413–446. doi:10.1016/S0140-6736(20)30367-6 32738937 PMC7392084

[2] Lo JC , Groeger JA, Cheng GH, Dijk DJ, Chee MW. Self-reported sleep duration and cognitive performance in older adults: a systematic review and meta-analysis. Sleep Med. 2016;17:87–98. doi:10.1016/j.sleep.2015.08.021 26847980

[3] Xu W , Tan C-C, Zou J-J, Cao X-P, Tan L. Sleep problems and risk of all-cause cognitive decline or dementia: an updated systematic review and meta-analysis. J Neurol Neurosurg Psychiatry. 2020;91(3):236–244. doi:10.1136/jnnp-2019-321896 31879285 PMC7035682

[4] Bubu OM , Brannick M, Mortimer J, et al. Sleep, cognitive impairment, and Alzheimer’s disease: a systematic review and meta-analysis. Sleep. 2016;40(1). doi:10.1093/sleep/zsw032 28364458

[5] Casagrande M , Forte G, Favieri F, Corbo I. Sleep quality and aging: a systematic review on healthy older people, mild cognitive impairment and Alzheimer’s Disease. Int J Environ Res Public Health. 2022;19(14):8457. doi:10.3390/ijerph19148457 35886309 PMC9325170

[6] Sabia S , Fayosse A, Dumurgier J, et al. Association of sleep duration in middle and old age with incidence of dementia. Nat Commun. 2021;12(1):2289. doi:10.1038/s41467-021-22354-2 33879784 PMC8058039

[7] Ohayon MM , Carskadon MA, Guilleminault C, Vitiello MV. Meta-analysis of quantitative sleep parameters from childhood to old age in healthy individuals: developing normative sleep values across the human lifespan. Sleep. 2004;27(7):1255–1273. doi:10.1093/sleep/27.7.1255 15586779

[8] Campbell SS , Murphy PJ. The nature of spontaneous sleep across adulthood. J Sleep Res. 2007;16(1):24–32. doi:10.1111/j.1365-2869.2007.00567.x 17309760

[9] Faubel R , LÓPez-GarcÍA E, Guallar-CastillÓN P, Graciani A, Banegas JR, RodrÍGuez-Artalejo F. Usual sleep duration and cognitive function in older adults in Spain. J Sleep Res. 2009;18(4):427–435. doi:10.1111/j.1365-2869.2009.00759.x 19691473

[10] Tworoger SS , Lee S, Schernhammer ES, Grodstein F. The association of self-reported sleep duration, difficulty sleeping, and snoring with cognitive function in older women. Physical & Somatoform & Psychogenic Disorders 3290. Alzheimer Dis Assoc Disord. 2006;20(1):41–48. doi:10.1097/01.wad.0000201850.52707.80 16493235

[11] Li Y , Sahakian BJ, Kang J, et al. The brain structure and genetic mechanisms underlying the nonlinear association between sleep duration, cognition and mental health. Nature Aging. 2022;2(5):425–437. doi:10.1038/s43587-022-00210-2 37118065

[12] Ma Y , Liang L, Zheng F, Shi L, Zhong B, Xie W. Association between sleep duration and cognitive decline. JAMA Netw Open. 2020;3(9):e2013573. doi:10.1001/jamanetworkopen.2020.13573 32955572 PMC7506513

[13] Liang Y , Qu LB, Liu H. Non-linear associations between sleep duration and the risks of mild cognitive impairment/dementia and cognitive decline: a dose-response meta-analysis of observational studies. Aging Clin Exp Res. 2019;31(3):309–320. doi:10.1007/s40520-018-1005-y 30039452

[14] Sexton CE , Storsve AB, Walhovd KB, Johansen-Berg H, Fjell AM. Poor sleep quality is associated with increased cortical atrophy in community-dwelling adults. Neurology. 2014;83(11):967–973. doi:10.1212/WNL.0000000000000774 25186857 PMC4162301

[15] Tai XY , Chen C, Manohar S, Husain M. Impact of sleep duration on executive function and brain structure. Commun Biol. 2022;5(1):201. doi:10.1038/s42003-022-03123-3 35241774 PMC8894343

[16] Murman DL. The impact of age on cognition. Semin Hear. 2015;36(3):111–121. doi:10.1055/s-0035-1555115 27516712 PMC4906299

[17] Goldman SE , Hall M, Boudreau R, et al. Association between nighttime sleep and napping in older adults. Sleep. 2008;31(5):733–740. doi:10.1093/sleep/31.5.733 18517043 PMC2398743

[18] Li P , Gao L, Yu L, et al. Daytime napping and Alzheimer’s dementia: a potential bidirectional relationship. Alzheimers Dement. 2023;19(1):158–168. doi:10.1002/alz.12636 35297533 PMC9481741

[19] Ancoli-Israel S , Martin JL. Insomnia and daytime napping in older adults. J Clin Sleep Med. 2006;02(03):333–342. doi:10.5664/jcsm.26597 17561549

[20] Cai H , Su N, Li W, Li X, Xiao S, Sun L. Relationship between afternoon napping and cognitive function in the ageing Chinese population. Gen Psychiatry. 2021;34(1):e100361. doi:10.1136/gpsych-2020-100361 PMC7839842 33585792

[21] Alqurashi YD , AlHarkan K, Aldhawyan A, et al. Association between nap duration and cognitive functions among Saudi older adults. Front Neurosci. 2022;16:917987. doi:10.3389/fnins.2022.917987 35720687 PMC9204222

[22] Kwak S , Kim H, Oh DJ, et al. Clinical and biological subtypes of late-life depression. J Affect Disord. 2022;312:46–53. doi:10.1016/j.jad.2022.06.005 35691418

[23] Shin C , Park MH, Lee SH, et al. Usefulness of the 15-item geriatric depression scale (GDS-15) for classifying minor and major depressive disorders among community-dwelling elders. J Affect Disord. 2019;259:370–375. doi:10.1016/j.jad.2019.08.053 31470180

[24] Becker NB , Jesus SN, João KADR, Viseu JN, Martins RIS. Depression and sleep quality in older adults: a meta-analysis. Psychol Health Med. 2017;22(8):889–895. doi:10.1080/13548506.2016.1274042 28013552

[25] Li XL , Wei J, Zhang X, Meng Z, Zhu W. Relationship between night-sleep duration and risk for depression among middle-aged and older people: a dose-response meta-analysis. Front Physiol. 2023;14:1085091. doi:10.3389/fphys.2023.1085091 36935736 PMC10017495

[26] McKinnon AC , Beath AP, Naismith SL. Relationships between sleep quality, depressive symptoms and MCI diagnosis: a path analysis. J Affect Disord. 2019;256:26–32. doi:10.1016/j.jad.2019.05.045 31158713

[27] Bertoux M , Lagarde J, Corlier F, et al. Sulcal morphology in Alzheimer’s disease: an effective marker of diagnosis and cognition. Neurobiol Aging. 2019;84:41–49. doi:10.1016/j.neurobiolaging.2019.07.015 31491594

[28] Lupton MK , Robinson GA, Adam RJ, et al. A prospective cohort study of prodromal Alzheimer’s disease: Prospective Imaging Study of Ageing: genes, brain and behaviour (PISA). Neuroimage Clin. 2021;29:102527. doi:10.1016/j.nicl.2020.102527 33341723 PMC7750170

[29] Sheikh JI , Yesavage JA: Geriatric Depression Scale (GDS): recent evidence and development of a shorter version. Clinical Gerontology: A Guide to Assessment and Intervention. NY: The Haworth Press; 1986: 165–173.

[30] Yesavage JA , Brink TL, Rose TL, et al. Development and validation of a geriatric depression screening scale: a preliminary report. J Psychiatr Res. 1983;17:37–49. doi:10.1016/0022-3956(82)90033-4 7183759

[31] Alden D , Austin C, Sturgeon R. A correlation between the Geriatric Depression Scale long and short forms. J Gerontol. 1989;44:P124–P125. doi:10.1093/geronj/44.4.p124 2738314

[32] Buysse DJ , Reynolds CF, Monk TH, Berman SR, Kupfer DJ. The Pittsburgh sleep quality index: a new instrument for psychiatric practice and research. Psychiatry Res. 1989;28(2):193–213. doi:10.1016/0165-1781(89)90047-4 2748771

[33] Borne L , Rivière D, Mancip M, Mangin J-F. Automatic labeling of cortical sulci using patch- or CNN-based segmentation techniques combined with bottom-up geometric constraints. Med Image Anal. 2020;62:101651. doi:10.1016/j.media.2020.101651 32163879

[34] Perrot M , Rivière D, Mangin JF. Cortical sulci recognition and spatial normalization. Med Image Anal. 2011;15(4):529–550. doi:10.1016/j.media.2011.02.008 21441062

[35] Hayes AF. Introduction to Mediation, Moderation, and Conditional Process Analysis: A Regression-Based Approach. New York: Guilford publications; 2017.

[36] Magnaldi S , Ukmar M, Vasciaveo A, Longo R, Pozzi-Mucelli R. Contrast between white and grey matter: MRI appearance with ageing. Eur Radiol. 1993;3:513–519.

[37] Spira AP , Gonzalez CE, Venkatraman VK, et al. Sleep Duration and subsequent cortical thinning in cognitively normal older adults. Sleep. 2016;39(5):1121–1128. doi:10.5665/sleep.5768 26951390 PMC4835311

[38] Lucey BP. It’s complicated: the relationship between sleep and Alzheimer’s disease in humans. Neurobiol Dis. 2020;144:105031. doi:10.1016/j.nbd.2020.105031 32738506 PMC7484285

[39] Spira AP , Gamaldo AA, An Y, et al. Self-reported sleep and β-amyloid deposition in community-dwelling older adults. JAMA Neurol. 2013;70(12):1537–1543. doi:10.1001/jamaneurol.2013.4258 24145859 PMC3918480

[40] Winer JR , Morehouse A, Fenton L, et al. Tau and b-amyloid burden predict actigraphy-measured and self-reported impairment and misperception of human sleep. J Neurosci. 2021;41(36):7687–7696. doi:10.1523/JNEUROSCI.0353-21.2021 34290080 PMC8425979

[41] Trifu SC , Trifu AC, Aluaş E, Tătaru MA, Costea RV. Brain changes in depression. Rom J Morphol Embryol. 2020;61(2):361–370. doi:10.47162/RJME.61.2.06 33544788 PMC7864313

[42] Liu X , Kakeda S, Watanabe K, et al. Relationship between the cortical thickness and serum cortisol levels in drug-naïve, first-episode patients with major depressive disorder: a surface-based morphometric study. Depress Anxiety. 2015;32(9):702–708. doi:10.1002/da.22401 26290363

[43] Lupien SJ , de Leon M, de Santi S, et al. Cortisol levels during human aging predict hippocampal atrophy and memory deficits. Nat Neurosci. 1998;1(1):69–73. doi:10.1038/271 10195112

[44] Lammer L , Beyer F, Luppa M, et al. Impact of social isolation on grey matter structure and cognitive functions: a population-based longitudinal neuroimaging study. eLife. 2023;12:e83660. doi:10.7554/eLife.83660 37337666 PMC10281670

[45] McDermott LM , Ebmeier KP. A meta-analysis of depression severity and cognitive function. J Affect Disord. 2009;119(1):1–8. doi:10.1016/j.jad.2009.04.022 19428120

[46] Silva GE , Goodwin JL, Sherrill DL, et al. Relationship between reported and measured sleep times: the Sleep Heart Health Study (SHHS). J Clin Sleep Med. 2007;3(6):622–630. 17993045 PMC2045712

[47] Jackson CL , Patel SR, Jackson WB 2nd, Lutsey PL, Redline S. Agreement between self-reported and objectively measured sleep duration among white, black, Hispanic, and Chinese adults in the United States: Multi-Ethnic Study of Atherosclerosis. Sleep. 2018;41(6). doi:10.1093/sleep/zsy057 PMC5995218 29701831

[48] Kessler RC , Berglund P, Demler O, et al.; National Comorbidity Survey Replication. The epidemiology of major depressive disorder results from the National Comorbidity Survey Replication (NCS-R). JAMA. 2003;289(23):3095–3105. doi:10.1001/jama.289.23.3095 12813115

