# Supplementary material for: A central role of sulcal width in the associations of sleep duration and depression with cognition in mid to late life
Source: Sleep Adv. 2024 Aug 10;5(1):zpae058. doi: 10.1093/sleepadvances/zpae058 (PMC11362672; doi:10.1093/sleepadvances/zpae058)
Supplement: zpae058_suppl_Supplementary_Tables_S1-S3_Figures_S1-S2 [file zpae058_suppl_supplementary_tables_s1-s3_figures_s1-s2.docx]

A Central Role of Sulcal Width in the Associations of Sleep Duration
and Depression with Cognition in Mid to Late Life

**Supplementary Materials**

*Faucher, C. ^1,2^, Borne, L.^1^ , Behler, A. ^1^, Paton, B.^1^, Giorgio, J.^1,3^, Fripp, J. ^2^, Thienel, R.^4^ ,*

*Lupton, M. K.^5#^, Breakspear, M. ^1,4#^*

^1^ School of Psychological Science, College of Science, Engineering and the Environment, University of Newcastle, Australia

^2^ Australian eHealth Research Centre, CSIRO, Brisbane, Australia

^3^Helen Wills Neuroscience Institute, University of California, Berkeley, USA

^4^ School of Public Health and Medicine, College of Health Medicine and Wellbeing, University of Newcastle, Australia

^5^ QIMR Berghofer Medical Research Institute

^*, #^Equally contributing

**Corresponding author**

Caroline Faucher

HMRI Imaging Centre, Lot 1 Kookaburra Circuit, New Lambton Heights, NSW, Australia, 2305

Email: caroline.faucher@uon.edu.au

**Supplementary Materials**

**Supplementary Figure 1**


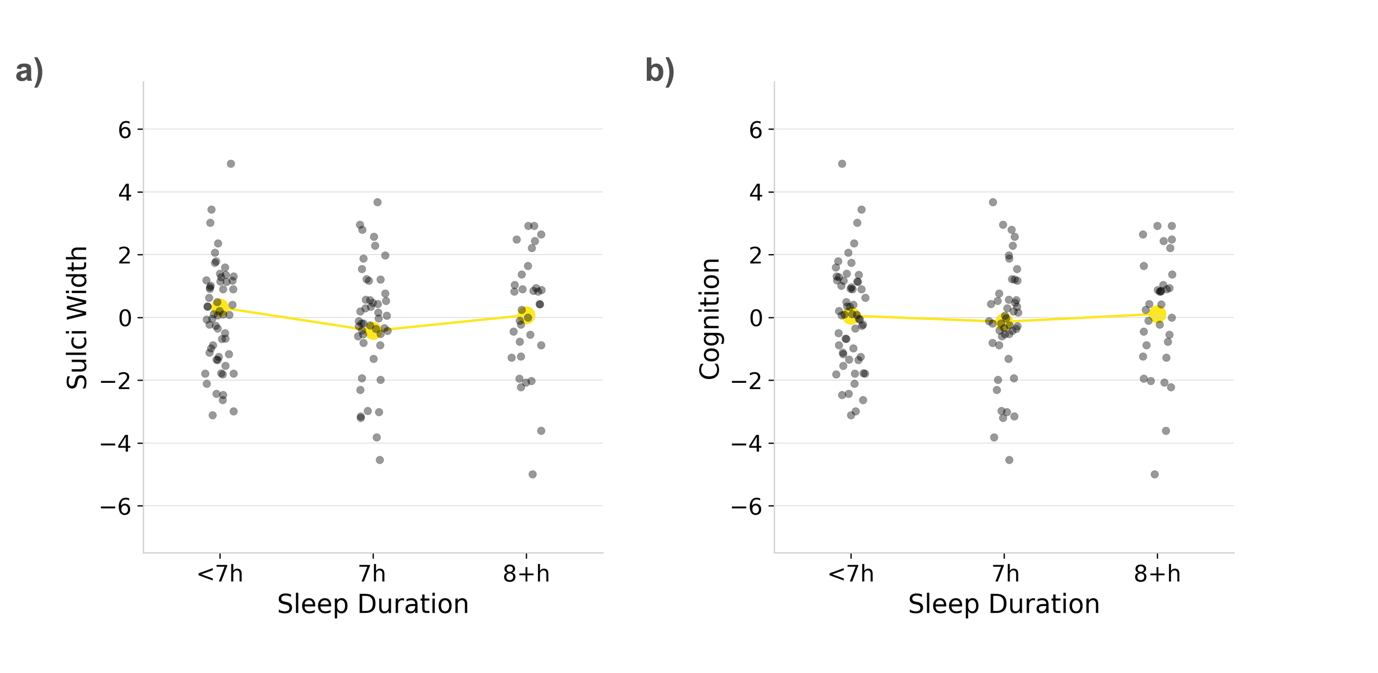


Sulci Width and Cognition Means by Sleep Duration. Visualization of means from sulci width composite variable (a) and cognition composite variable (b) based on self-reported sleep durations, showing short to long durations.

**Supplementary Figure 2**





Atlas of Sulci with Nomenclature TableLocation of sulci named by their Brainvisa acronyms or their full anatomical name (see Perrot et al., 2011).

**Supplementary Table 1**. Results from additional ANCOVA with Overnight Sleep Duration Factor

| Dependent variable | Source | F | *P* | Partial η² |
| --- | --- | --- | --- | --- |
| Cognition  (covariates: age, sex) | Sleep Duration | 1.46 | .237 | 0.02 |
| Sulci Width  (covariates: age, sex) | Sleep Duration | 2.07 | .130 | 0.03 |

*Note.* *p ≤ .05. Sleep duration factor segmented into three categories: <7 hours, 7 hours, ≥8 hours.

**Supplementary Table 2**. Estimated Marginal Means for additional ANCOVA with Overnight Sleep Duration Factor

| Dependent variable | Sleep Duration | Mean | Std. Error | CI |
| --- | --- | --- | --- | --- |
| Cognition  (covariates: age, sex) |  |  |  |  |
|  | <7 hours | 0.18 | 0.20 | [-0.23,0.58] |
|  | 7 hours | -0.31 | 0.23 | [-0.76,0.13] |
|  | ≥8 hours | 0.14 | 0.27 | [-0.39,0.67] |
| Sulci Width  (covariates: age, sex) |  |  |  |  |
|  | <7 hours | 0.48 | 0.48 | [-0.47,1.43] |
|  | 7 hours | -0.88 | 0.53 | [-1.93,0.17] |
|  | ≥8 hours | 0.43 | 0.63 | [-0.83,1.67] |

*Note.* *p ≤ .05. 95% Confidence Interval (CI). Sleep duration factor segmented into three categories:
<7 hours, 7 hours, ≥8 hours.

**Supplementary Table 3**. Additional Results from ANCOVA Analysis with APOE ɛ4 Factor

| Dependent variable | Source | F | *P* | Partial η² |
| --- | --- | --- | --- | --- |
| Cognition  (covariates: age, sex) | APOE ɛ4 status | 0.58 | .449 | 0.004 |
| Sulci Width  (covariates: age, sex) | APOE ɛ4 status | 8.460x10^-4^ | .977 | 6.361x10^-6^ |

*Note.* *p ≤ .05, APOE ɛ4 status is defined as positive if the participant had at least one allele.

**Appendix A**

Creyos Online Cognitive Tests (formerly Cambridge Brain Sciences)

| Test | Description* |
| --- | --- |
| Digit Span  (Verbal short-term memory) | Digit Span measures verbal short-term memory, defined as the system that allows for temporary storage of information, and is crucial in everyday tasks such as remembering a telephone number or understanding long sentences. Digit Span involves numbers, but performance is indicative of verbal short-term memory, because it requires dealing with items in a specific order, as opposed to spatial short-term memory. |
| Double Trouble  (Response inhibition) | Double trouble (based upon the Stroop task) assesses response inhibition, which is the ability to concentrate on relevant information to make an appropriate response, even when distracting information or interference is present. It is a key component of concentration. |
| Feature Match (Attention) | A task measuring attention—the ability to muster mental resources to focus and monitor for a specific stimulus or difference. Identifying similarities and differences is an important real-life skill that is put to the test in this difficult version of “spot the difference.” |
| Grammatical  Reasoning (Verbal reasoning) | Verbal reasoning is the ability to quickly understand and make valid conclusions about concepts expressed in words. While language comes naturally to most people, understanding complex sentences with multiple negative statements is consistently challenging. |
| Monkey Ladder (Visuospatial working memory) | Monkey ladder assesses visuospatial working memory, which is the ability to not only hold information in memory, but manipulate or update it based on changing circumstances. Monkey Ladder requires storing numbers and their locations, then translating that memory into a series of movements in space. |
| Odd One Out (Deductive reasoning) | Odd One Out assesses deductive reasoning, which is the core cognitive ability to apply rules to information in order to arrive at a logical conclusion. Odd One Out requires reasoning about the features of several shapes to deduce the one shape that does not fit in with the rest. |
| Paired Associates  (Episodic memory) | Episodic memory is the ability to remember and recall specific events, paired with the context in which they occurred. Our Paired Associates assesses episodic memory by asking patients to remember which objects they previously saw, along with the location where they were seen. |
| Polygons (Visuospatial processing) | Polygons assesses visuospatial processing, which is the ability to effectively interpret visual information, such as complex visual stimuli and relationships between objects. Polygons challenges the patient’s proficiency in picking out subtle differences between shapes. |
| Rotations (Mental rotation) | Mental rotation is a function of visual representation in the brain. Effectively manipulating mental representations of objects allows people to make valid conclusions about what objects are and where they belong. |
| Spatial Planning | Planning is a fundamental property of intelligent behaviour. Spatial Planning assesses the patient’s ability to act with forethought and sequence behaviour in an orderly fashion to reach specific goals. |
| Spatial Span  (Planning) | Spatial short-term memory is the cognitive system allowing for temporary storage of spatial information. Spatial Span challenges the patient’s ability to remember the relationships between objects in space, as opposed to verbally rehearsing items in specific order, which relies on verbal short-term memory. |
| Token Search (Working memory) | Working memory is the ability to temporarily hold information in memory, and manipulate it based on changing circumstances or demands. In Token Search, patients need to maintain and update an ongoing representation of previous searches in a self-directed task. |

*Note* *Descriptions reproduced from Creyos.com
